# Human Metapneumovirus: Emergence, Impact, and Public Health Significance

**DOI:** 10.7759/cureus.80964

**Published:** 2025-03-21

**Authors:** Laresh N Mistry, Sumeet Agarwal, Himmat Jaiswal, Saba Kondkari, Sayem A Mulla, Sankalp D Bhandarkar

**Affiliations:** 1 Pedodontics and Preventive Dentistry, Bharati Vidyapeeth (Deemed to be University) Dental College and Hospital, Navi Mumbai, IND; 2 Prosthodontics, Bharati Vidyapeeth (Deemed to be University) Dental College and Hospital, Navi Mumbai, IND; 3 Conservative Dentistry and Endodontics, Bharati Vidyapeeth (Deemed to be University) Dental College and Hospital, Navi Mumbai, IND; 4 Dentistry, Bharati Vidyapeeth (Deemed to be University) Dental College and Hospital, Navi Mumbai, IND; 5 Dentistry, Dr. N. Y. Tasgaonkar Institute of Medical Science, Raigad, IND

**Keywords:** acute respiratory syndrome, chronic disease epidemiology, hmpv, human metapneumovirus (hmpv), paramyxoviridae, public health care, respiratory viruses, severe acute respiratory infection

## Abstract

Human metapneumovirus (hMPV) has re-emerged as a significant respiratory pathogen in recent times and has attracted significant attention worldwide. Initially, identified in children with respiratory infections with significant impact, hMPV has been implicated for its contribution to global respiratory illness. The unique features of this virus, its origin, evolution, and epidemiological importance has been explored in this narrative review. Additionally, it discusses factors contributing to its recent recognition, including advancements in diagnostic methods, its clinical impact, and public health implications.

## Introduction and background

Respiratory tract infections (RTIs) are among the leading causes of morbidity and mortality worldwide. Numerous significant infections affecting both humans and animals belong to the Paramyxovirinae and Pneumovirinae subfamilies of the Paramyxoviridae family. The Pneumovirus and Metapneumovirus genera constitute the taxonomic classifications of the Pneumovirinae subfamily. Prior to 2001, Metapneumoviruses had not been linked to infections or diseases in mammals [1]. Human metapneumovirus (hMPV) has since emerged as a major pathogen, alongside established respiratory viruses like influenza, respiratory syncytial virus (RSV), and coronaviruses. Initially identified in the early 21st century, hMPV is now recognized for causing severe respiratory infections, particularly in at-risk populations, including children, the elderly, and immunocompromised individuals. Researchers identified the virus from nasopharyngeal aspirate samples of 28 epidemiologically unrelated children with RTIs in the Netherlands between 1980 and 1999. Thirteen of these patients were infants aged 0-12 months, and 27 were under five years old [2]. This article examines the discovery, epidemiology, and clinical significance of hMPV, along with the factors leading to its increased attention in recent research and discussion in the scientific community.

Discovery and evolution

Origin

hMPV is a member of the Paramyxoviridae family, which also includes RSV, measles, and mumps viruses. It was initially isolated in the Netherlands in 2001 from children with respiratory symptoms [1]. Genetic studies suggest that hMPV likely diverged from avian metapneumovirus approximately 200 years ago. Serological research shows that hMPV was circulating in the Netherlands as early as 1958, but its role in clinical symptoms in children was not recognized until its discovery in 2001 [3]. Despite its relatively recent identification, retrospective analyses have detected its presence in archival samples dating back several decades.

Structure and Genomic Features

**Figure 1:**
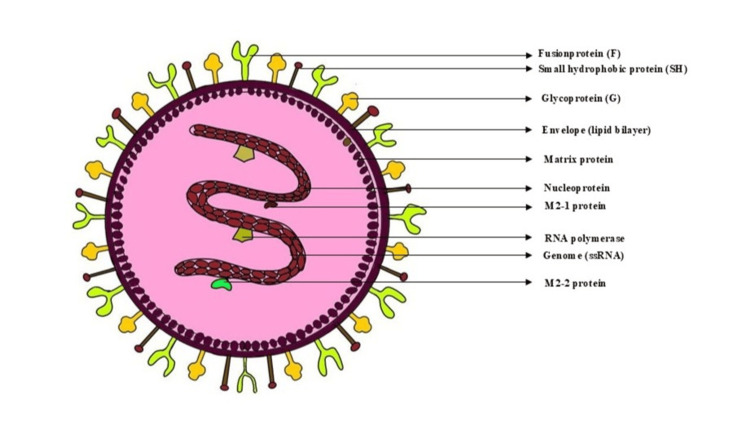
Structure and genomic features of human metapneumovirus. Image credits: Dr. Sayem A Mulla, Dr. Saba Kondkari, Dr. Laresh N Mistry.

The hMPV genome is a single-stranded, negative-sense RNA approximately 13 kb in length. It encodes nine proteins, including the fusion (F) and attachment (G) glycoproteins essential for host cell entry and immune evasion. Two principal genotypes, A and B, along with several sublineages, contribute to the virus's genetic diversity and periodic outbreaks. hMPV is linked to a range of respiratory illnesses, varying from mild upper RTIs, such as the common cold, to severe lower respiratory tract conditions, including bronchitis, pneumonia, and asthma exacerbations. Severe manifestations are more frequent in young children, elderly individuals, and immunocompromised patients. However, severe infections have also been observed in healthy adults and elderly individuals with comorbidities (Figure 1) [4].

Epidemiology and Transmission

hMPV has been detected globally, with peak activity occurring in late winter and early spring in temperate climates [5]. It is estimated to account for 4-7% of acute respiratory infections (ARIs) in adults and 5-10% in children. Studies indicate that hMPV infection rates are comparable to those of RSV and influenza in certain populations, underlining its epidemiological significance. hMPV affects individuals of all age groups; however, they are most prevalent among pediatric patients [6]. The first hMPV infection typically occurs around six months of age, following which recurrent infections may follow. The elderly represent the second most affected group, experiencing severe infections despite high seroprevalence rates, which appear unaffected by immunosenescence. Reports of hMPV infections in otherwise healthy individuals are relatively uncommon [6]. Sentinel general practice locations in Australia provide some hMPV data despite the lack of a nationwide collection. A summary of the data shows that 7.8% of patients with coughing and high temperature who underwent respiratory pathogen testing in 2024 (up until December 15) had hMPV [7]. Over 15 cases have been recorded in India, with the initial instances found in Bengaluru. On the same day, a case was recorded in Gujarat, followed by two confirmed cases in Nagpur, Maharashtra. Since then, additional cases have been reported in Gujarat, Ahmedabad, Pondicherry, and Assam [8].

**Figure 2:**
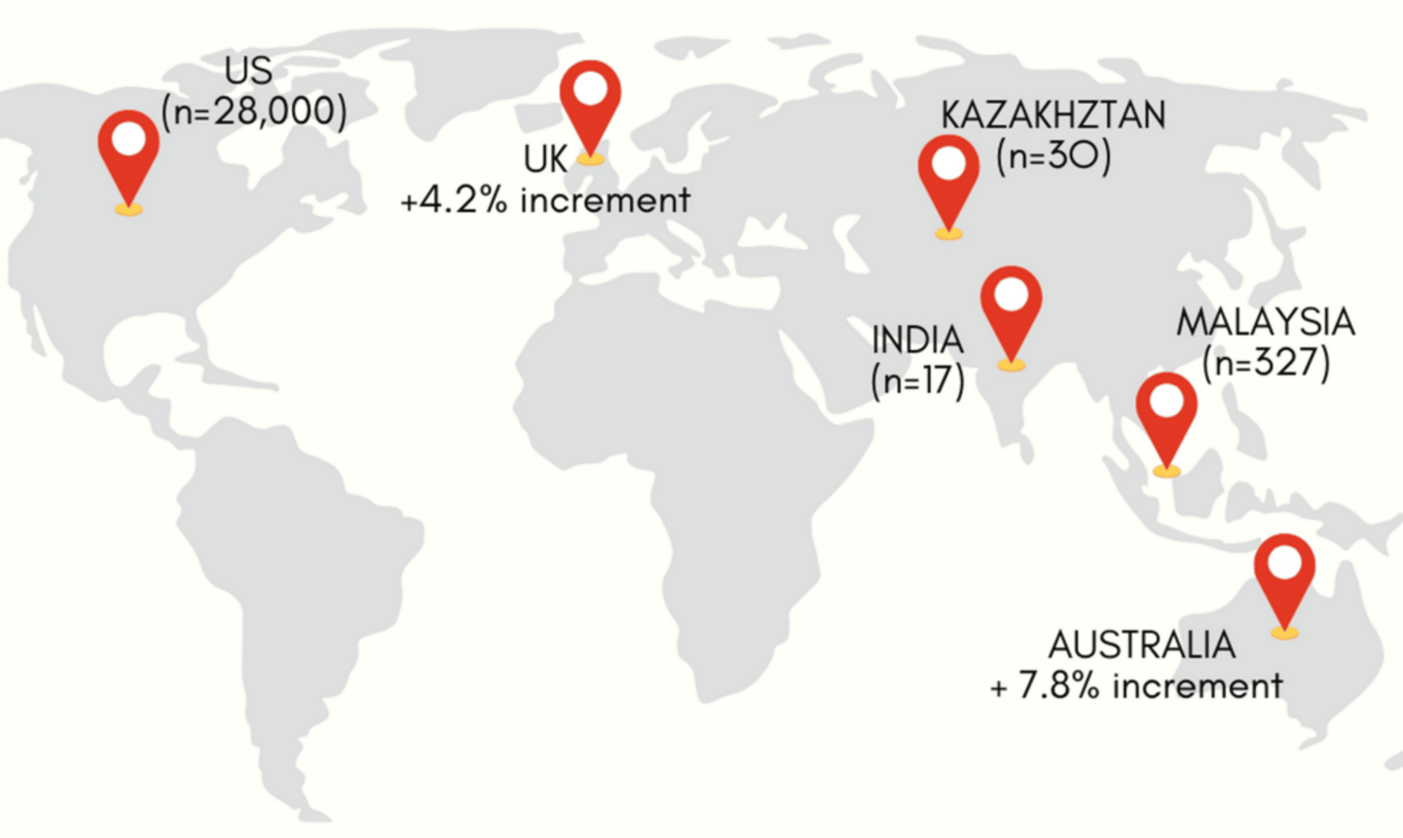
Global human metapneumovirus data from January 2024 to January 2025. Image credits: Dr. Saba Kondkari, Dr. Sayem A Mulla, Dr. Laresh N Mistry.

The Republic of Kazakhstan's Ministry of Health has also documented cases as part of the anticipated annual increase in flu and other respiratory infections. Compared to other respiratory illnesses, the current flu season has shown 30 instances of hMPV, according to the Committee for Sanitary and Epidemiological Control [9]. Malaysia has witnessed a notable increase in HMPV cases, with reports indicating 327 cases in 2024- a 45% increase from 225 cases in 2023 [10]. Weekly Centers for Disease Control and Prevention (CDC) statistics since January 2024 indicate that nearly 28,000 Americans have contracted the virus. Similarly, the latest data from the UK Health Security Agency (UKHSA) reveal a 4.15 percentage point increase in hMPV cases between October and December of last year (Figure 2) [11].

Modes of Transmission

The virus primarily spreads through close contact between individuals, respiratory droplets, and direct contact with contaminated surfaces. Its incubation period ranges from four to six days, with viral shedding lasting up to two weeks in immunocompetent individuals and extending further in immunocompromised patients [12]. The F protein on the surface of virus particles is believed to be unnecessary for hMPV to transmit directly from one cell to another in the presence of neutralizing antibodies and the absence of attachment factors required for virus particle entry into cells [13]. According to surveillance data gathered from the CDC's National Respiratory and Enteric Virus Surveillance System, the virus appears to be most active in temperate regions during the late winter and spring. Throughout the respiratory virus season, influenza, RSV, and hMPV circulate concurrently [14].

## Review

Clinical manifestations

Symptoms and Severity

Clinical features observed in young individuals, especially children, include moderate upper respiratory tract illness as well as severe bronchiolitis and pneumonia, comparable to those induced by RSV infection. Very young children infected with hMPV often need hospitalization and mechanical ventilation, similar to those with RSV [1]. hMPV primarily affects both the upper and lower respiratory tracts, resulting in a range of illnesses from mild colds to severe bronchiolitis and pneumonia. Frequently observed symptoms include fever, cough, nasal congestion, and wheezing. In severe instances, hypoxia, respiratory distress, and hospitalization may be required, particularly among young children, the elderly, and individuals with chronic health conditions.

Risk Factors

In 2012, hMPV and RSV were the leading pediatric pathogens worldwide [15]. Prematurity, female sex, and genotype B infection were identified as risk factors for severe hMPV illness among hospitalized patients. While breastfeeding and viral coinfection provided protection against RSV hospitalization, risk factors included age under six months, comorbidities, and household crowding [15].

Immune Response to hMPV

Understanding the immunopathology of hMPV infection and the development of effective vaccines or therapies necessitates a thorough examination of the host immune response. hMPV generates a partial memory response and, like other viruses, interacts with the host's immune system through both innate and adaptive mechanisms [16,17]. The virus interacts with immune cells in the respiratory tract, and the protection against it is mediated by pattern recognition receptors and pathogen-associated molecular patterns. These include C-type lectin receptors, nucleotide-binding oligomerization domain-like receptors, cytoplasmic RIG-I-like receptors, and membrane-bound toll-like receptors (TLRs). Inflammation triggered by the immune system upon identifying viral molecular patterns occurs through cytokine production by respiratory cells, specifically via the MyD88 receptor-mediated pathway (a form of TLR) [18,19]. However, the viral G protein is known to inhibit the innate response in the cell cytoplasm by targeting RIG-I [20,21].

While humoral immunity does not target the G and SH proteins, it is effective against the F protein [22]. Cytotoxic T lymphocytes mediate the cell-mediated response by inducing apoptosis in infected cells or activating Th cells, which subsequently activate B cells and other immune cells, including macrophages and dendritic cells [19]. This is evident from the identification of virus-specific T lymphocytes in the lungs seven days after infection. However, in later stages, pulmonary damage is primarily attributed to a Th2-skewed response [23-26]. This is also controlled due to a reduction in CD4+ T cells, which initially lowers disease severity, after which CD8+ T cells eliminate the residual infection [25]. However, CD8+ T cells show a diminishing response due to the increased expression of programmed death-1 and programmed death ligand-1, leading to suppression of activity of these cells [27-29].

An immunocompetent individual reacts to viral infection with an antiviral immune response; however, the virus evades the immunological defense utilizing virus-specific mechanisms. In contrast to RSV, hMPV lacks nonstructural proteins and instead employs its M2-2 proteins-primarily targeting through mitochondrial antiviral signaling protein and reducing tumor necrosis factor receptor-associated factors-along with G and SH proteins to avoid the innate immune defense [21,30]. Many seroepidemiological studies have demonstrated that nearly all individuals are infected with hMPV before age five; however, recurrent infections occur throughout life, possibly due to waning host immunity to viral antigens and the lack of cross-reactive antibodies [31].

Diagnostic techniques

RT-PCR

RT-PCR (MM1) tests have been used extensively for viral molecular detection, particularly for hMPV, in recent decades. Typically, the F and N genes-two genomic areas with high sequence similarity in hMPV-are commonly utilized as molecular markers for developing RT-PCR techniques and can also be utilized for genotyping analysis. The mean operation time is around 3 to 5 hours, with the ability to detect 1,000 copies per reaction, and is cost-effective [32,33]. In general, RT-PCR tests for pathogen identification are less sensitive than RT-qPCR techniques, which need more sophisticated equipment. As a result, RT-PCR has been used less often in recent years for hMPV clinical identification [34].

RT-qPCR

RT-qPCR has been widely used for viral nucleic acid detection due to its high sensitivity, accuracy, and efficiency. As the gold standard diagnostic technique, RT-qPCR often offers greater sensitivity and a reduced chance of contamination than traditional RT-PCR techniques. In 2008, a TaqMan-based RT-qPCR method was developed, achieving a limit of detection (LOD) of 10 copies/μL. Using this method, 19.62% (31/158) of clinical samples tested positive for hMPV, whereas only 13.92% (22/158) were confirmed positive by traditional RT-PCR [35]. Sugimoto et al. created a duplex RT-qPCR assay for hMPV detection, demonstrating high sensitivity (<10 copies/reaction) for identifying and differentiating between hMPV A and B subgroups [36].

Multiplex RT-qPCR techniques have been developed for the synchronized detection of hMPV and other pathogens associated with acute RTIs, as numerous viruses cause these infections. You et al. introduced a reliable triple TaqMan-based RT-qPCR assay for the detection of hMPV, RSV, and the internal control gene (GAPDH), with a LOD of 100 copies/reaction for both hMPV and RSV [35]. Furthermore, Feng et al. developed a multiplex one-tube nested RT-qPCR assay capable of distinguishing RSV, HRV, and hMPV [37], achieving an analytical sensitivity of 5 copies/reaction for all three infections. The method is cost-effective and requires approximately one to three hours.

Besides, digital microfluidic (DMF) technology has been increasingly used in several areas, including pathogen diagnostics [38]. For the multiple detection of eleven respiratory infections, including hMPV, Huang et al. created an RT-qPCR platform in conjunction with DMF technology. The off-chip and on-chip RT-qPCR methods demonstrated sensitivities of ≤150 copies/reaction and ≤120 copies/reaction, respectively, for pathogen identification [39].

LAMP Technology

LAMP technology, one of the most widely used isothermal techniques for pathogen identification, was initially developed in 2000 by a Japanese team [40]. Song et al. designed two RT-LAMP reactions to identify and distinguish hMPV genotypes A and B, respectively. Using the online LAMP primer design tool Primer-Explorer V4, they developed four pairs of primers targeting the M genes [41]. The developed approach showed a tenfold higher sensitivity than traditional RT-PCR methods, with an LOD of 4.33 copies/μL for hMPV genotype A and 3.53 copies/μL for genotype B. In a similar vein, Wang et al. created three primer pairs targeting the N gene for hMPV detection. The developed method demonstrated superior sensitivity, detecting less than 10 copies/μL, outperforming the RT-PCR method [42].

When combined, LAMP achieves high amplification efficiency in an isothermal environment (~65°C) without the need for sophisticated equipment for temperature change. The use of two or three pairs of primers further increases amplification efficiency, contributing to the approach's high specificity. Additionally, LAMP detection findings are visible to the unaided eye, simplifying the detection process [43]. However, the process takes around 1.5 hours but is relatively expensive.

Recombinase-aided Amplification

Recombinase-aided amplification (RAA) is a recently developed thermostatic amplification technique widely used for disease diagnosis. It features simple equipment, uncomplicated operating procedures, and a high amplification efficiency. In order to develop the RT-RAA technique, Jiao et al. designed primers targeting the conservative region of the hMPV N gene. The method attained a LOD of 100 copies/μL, which was much lower than that of the commercial RT-qPCR approach [44] Additionally, the RT-RAA reaction system required a shorter running time, operating at 39 °C for 15 min.

CRISPER-Cas12a

Clustered regularly interspaced short palindromic repeats (CRISPR) and CRISPR-associated (Cas) proteins have been extensively used in gene editing [45]. Three kinds of Cas protein families have been identified based on the genetic traits of Cas genes in prokaryotes [46]. Among them, the CRISPR-Cas12a system is regarded as a potent tool for in vitro nucleic acid detection [47]. Additionally, integrating the CRISPR-Cas12a detection system with reverse transcription and isothermal amplification technologies like LAMP [48], RAA [49], and recombinase polymerase amplification (RPA) [50] could significantly increase its sensitivity and resilience. Moreover, when paired with lateral flow (LF), the detection result becomes visible to the naked eye, making this technology suitable for point-of-need locations such as airports and customs facilities. Qian et al. effectively developed a method for detecting hMPV RNA by fusing the CRISPR-Cas12a system with RT-RPA. In short, RT-RPA was used to amplify the hMPV N gene, while CRISPR-Cas12a identified the resulting products in conjunction with LF. With a LOD below 700 copies/mL, the RT-RPA and Cas12a-based LF test was completed within 30 minutes. Furthermore, the well-established RT-RPA-Cas12a-LF technique demonstrated a 96.4% detection concordance with the quantitative RT-PCR test, suggesting its potential as a viable substitute for hMPV diagnosis in settings lacking specialized equipment [51].

Metagenomic Next-Generation Sequencing

Metagenomic next-generation sequencing (mNGS), a high-throughput diagnostic technique, has been widely used for novel pathogen detection, viral genome sequencing, and other related research. Traditional detection techniques such as RT-PCR and RT-qPCR may not be able to detect emerging diseases, whereas mNGS can detect new or previously undiscovered pathogens [52]. Additionally, mNGS enables highly precise amplification of entire viral genomes. However, the RNA genome needs to be reverse-transcribed into complementary DNA before sequencing using platforms such as Illumina and Sanger sequencing. Xu et al. investigated the nosocomial transmission of HMPV among hematological patients using nanopore metagenomic sequencing technology [38]. This technique produced hMPV reads from 80% (20/25) of the samples and retrieved complete hMPV genomes from 15 samples. While the sensitivity of this method requires further improvement, it shows potential for diagnosing HMPV infections [53]. However, its high cost and prolonged processing time (5−10 days) remain significant limitations.

Virus Isolation

Viral isolation from clinical samples serves as the gold standard for pathogen detection and represents the first step in vaccine development and viral pathogenesis research [54]. The LLC-MK2 cell line has been commonly utilized in hMPV isolation and culture; however, the human Chang conjunctiva cell line (clone 1-5C4), LLC-MK2 cell line, and feline kidney CRFK cell line are also generally acceptable for hMPV isolation [55,56]. Viral isolation procedures have two primary processes: treating a cell monolayer with the supernatant of ana hMPV-positive samples and detecting hMPV antigens or nucleic acids using molecular or serological detection techniques 48-96 hours post-infection. Since hMPV grows slowly in cell lines and cytopathic effects are rarely seen, serological/molecular tests are commonly utilized to identify hMPV antigens/nucleic acids [56].

Recent spotlight on hMPV

Advances in Diagnostic Techniques

The growing utilization of multiplex polymerase chain reaction (PCR) panels has significantly improved the detection of various viruses attacking the respiratory system, including hMPV. This technology allows the simultaneous identification of multiple pathogens, facilitating accurate epidemiological assessments. PCR assays remain the most widely used and reliable approach for detecting hMPV, with multiplex RT-PCR being the most common (57.4%), followed by real-time RT-PCR (38.3%) [56].

Public Health Implications

The COVID-19 pandemic highlighted the burden of respiratory viruses, prompting increased surveillance and awareness of pathogens like hMPV. With increased focus on respiratory health, the substantial role of hMPV in hospitalizations and healthcare costs became evident. Patients with hematologic malignancies may face fatal outcomes from hMPV infection [57].

Outbreak Reports and Media Coverage

Recent outbreaks in nursing homes, daycare centers, and hospitals have underscored hMPV's capacity for rapid transmission in enclosed environments. These events have garnered media attention, emphasizing the need for targeted prevention and control strategies.

Prevention

The CDC has outlined specific preventive measures for hMPV. Hands should be washed for at least 20 seconds, and unwashed hands should not touch the eyes, nose, and/or mouth. Close contact with infected individuals should be temporarily avoided. Those exhibiting cold-like symptoms should be encouraged to take self-precautionary measures. Wash hands appropriately and frequently with soap and water for a minimum of 20 seconds. Avoid sharing cups and other eating utensils with others. Refrain from close contact, such as kissing, and stay home if you feel unwell. Cleanall possible contaminated surfaces, such as shared items and doorknobs. In healthcare settings, follow the CDC's 2007 guidelines for isolation precautions [14].

Vaccine development

As of January 2024, a new multi-epitope mRNA vaccine candidate designed to combat hMPV using immunoinformatics techniques was developed [58].

Antiviral therapies

Currently, no specific antiviral treatments are available for hMPV. In severe cases, management predominantly relies on supportive care, including oxygen therapy and mechanical ventilation. Experimental therapies, such as monoclonal antibodies and fusion inhibitors, are under investigation. Supportive interventions remain the cornerstone of treatment, with antipyretic drugs such as acetaminophen and ibuprofen prescribed for fever. Intravenous fluid hydration is recommended for patients who appear dehydrated and cannot handle oral hydration.

Furthermore, individuals with hMPV may need additional oxygen support, such as a high-flow nasal cannula or mechanical ventilation in extreme situations, resulting in acute respiratory failure. This is particularly relevant for patients with pre-existing respiratory or cardiac conditions or those who are immunocompromised. While most individuals eventually achieve full recovery, all hMPV patients should be placed on droplet precautions to limit and prevent transmission [3].

Future directions

Combining various nanotechnology with cutting-edge genetic platforms like next-generation sequencing holds significant potential for effective treatment interventions, offering substantial benefits to patients and the general population. However, analytical and clinical validation are necessary before implementing these novel procedures in clinical laboratories [56].

Public health interventions

Measures to control the spread of infection, such as following necessary hand hygiene, mask use, and isolating symptomatic individuals, remain vital for preventing hMPV transmission. Educational campaigns can raise awareness and help reduce the stigma associated with respiratory infections.

## Conclusions

hMPV is an important respiratory pathogen with a global impact. It has been increasingly recognized for its role in respiratory illness across all age groups with varying severity and epidemiological impacts. Advances in diagnostic techniques and growing awareness have brought hMPV into focus, and addressing the challenges in vaccine development, antiviral therapies, and surveillance is key to reducing its burden.

## References

[1] van den Hoogen BG, de Jong JC, Groen J, Kuiken T, de Groot R, Fouchier RA, Osterhaus AD (2001). A newly discovered human pneumovirus isolated from young children with respiratory tract disease. Nat Med.

[2] Lamb RA, Parks GD (2007). Paramyxoviridae: The viruses and their replication. Fields virology.

[3] Uddin S, Thomas M Human Metapneumovirus. StatPearls: Treasure Island.

[4] Costa-Filho RC, Saddy F, Costa JL, Tavares LR, Castro Faria Neto HC (2025). The silent threat of human metapneumovirus: Clinical challenges and diagnostic insights from a severe pneumonia case. Microorganisms.

[5] Kahn JS (2006). Epidemiology of human metapneumovirus. Clin Microbiol Rev.

[6] Schildgen V, van den Hoogen B, Fouchier R (2011). Human metapneumovirus: Lessons learned over the first decade. Clin Microbiol Rev.

[7] (2025). Australia monitoring international increases in human metapneumovirus (hMPV). https://www.cdc.gov.au/newsroom/news-and-articles/australia-monitoring-international-increases-human-metapneumovirus-hmpv.

[8] (2025). HMPV virus cases in India live updates: four-year-old tests positive for HMPV in Ahmedabad, state tally reaches six. https://www.onlymyhealth.com/hmpv-virus-cases-live-updates-check-health-ministry-guidelines-state-wise-and-cases-of-human-metapneumovirus-12977823422.

[9] HMPV Outbreak: Full List of Countries With Reported Cases. Newsweek (2025). HMPV outbreak: Full list of countries with reported cases. http://newsweek.com/hmpv-outbreak-countries-china-malaysia-india-kazakhstan-2010210.

[10] (2025). HMPV tracker: Are infections surging in India, China and the UK?. https://www.firstpost.com/health/hmpv-tracker-how-many-cases-in-india-china-malaysia-uk-13850575.html.

[11] Toth A (2025). Everything we know about HMPV in the UK: Symptoms, case numbers and how to get a test. https://www.msn.com/en-nz/health/other/everything-we-know-about-hmpv-in-the-uk-symptoms-case-numbers-and-how-to-get-a-test/ar-AA1x3kUm.

[12] Williams JV, Harris PA, Tollefson SJ (2004). Human metapneumovirus and lower respiratory tract disease in otherwise healthy infants and children. N Engl J Med.

[13] El Najjar F, Castillo SR, Moncman CL (2023). Imaging analysis reveals budding of filamentous human metapneumovirus virions and direct transfer of inclusion bodies through intercellular extensions. mBio.

[14] Human Metapnemovirus. CDC (2025). Human metapnemovirus. https://www.cdc.gov/human-metapneumovirus/about/index.html.

[15] Papenburg J, Hamelin MÈ, Ouhoummane N (2012). Comparison of risk factors for human metapneumovirus and respiratory syncytial virus disease severity in young children. J Infect Dis.

[16] Douville RN, Bastien N, Li Y, Pochard P, Simons FE, HayGlass KT (2006). Human metapneumovirus elicits weak IFN-gamma memory responses compared with respiratory syncytial virus. J Immunol.

[17] González AE, Lay MK, Jara EL (2017). Aberrant T cell immunity triggered by human respiratory syncytial virus and human metapneumovirus infection. Virulence.

[18] Cheemarla NR, Guerrero-Plata A (2015). Immune response to human metapneumovirus infection: What we have learned from the mouse model. Pathogens.

[19] Ren J, Kolli D, Deng J (2013). MyD88 controls human metapneumovirus-induced pulmonary immune responses and disease pathogenesis. Virus Res.

[20] Bao X, Liu T, Shan Y, Li K, Garofalo RP, Casola A (2008). Human metapneumovirus glycoprotein G inhibits innate immune responses. PLoS Pathog.

[21] Kolli D, Bao X, Casola A (2012). Human metapneumovirus antagonism of innate immune responses. Viruses.

[22] Falsey AR, Hennessey PA, Formica MA, Criddle MM, Biear JM, Walsh EE (2010). Humoral immunity to human metapneumovirus infection in adults. Vaccine.

[23] Alvarez R, Tripp RA (2005). The immune response to human metapneumovirus is associated with aberrant immunity and impaired virus clearance in BALB/c mice. J Virol.

[24] Herd KA, Nelson M, Mahalingam S, Tindle RW (2010). Pulmonary infection of mice with human metapneumovirus induces local cytotoxic T-cell and immunoregulatory cytokine responses similar to those seen with human respiratory syncytial virus. J Gen Virol.

[25] Kolli D, Bataki EL, Spetch L (2008). T lymphocytes contribute to antiviral immunity and pathogenesis in experimental human metapneumovirus infection. J Virol.

[26] Darniot M, Pitoiset C, Petrella T, Aho S, Pothier P, Manoha C (2009). Age-associated aggravation of clinical disease after primary metapneumovirus infection of BALB/c mice. J Virol.

[27] Erickson JJ, Gilchuk P, Hastings AK (2012). Viral acute lower respiratory infections impair CD8+ T cells through PD-1. J Clin Invest.

[28] Erickson JJ, Rogers MC, Hastings AK, Tollefson SJ, Williams JV (2014). Programmed death-1 impairs secondary effector lung CD8⁺ T cells during respiratory virus reinfection. J Immunol.

[29] Wen SC, Schuster JE, Gilchuk P, Boyd KL, Joyce S, Williams JV (2015). Lung CD8+ T cell ompairment occurs during human metapneumovirus infection despite virus-like particle induction of functional CD8+ T cells. J Virol.

[30] Chen Y, Deng X, Deng J (2016). Functional motifs responsible for human metapneumovirus M2-2-mediated innate immune evasion. Virology.

[31] Kumar P, Srivastava M (2018). Prophylactic and therapeutic approaches for human metapneumovirus. Virusdisease.

[32] Cong S, Wang C, Wei T (2022). Human metapneumovirus in hospitalized children with acute respiratory tract infections in Beijing, China. Infect Genet Evol.

[33] Wang C, Wei T, Ma F (2021). Epidemiology and genotypic diversity of human metapneumovirus in paediatric patients with acute respiratory infection in Beijing, China. Virol J.

[34] Li J, Mao NY, Zhang C, Yang MJ, Wang M, Xu WB, Ma XJ (2012). The development of a GeXP-based multiplex reverse transcription-PCR assay for simultaneous detection of sixteen human respiratory virus types/subtypes. BMC Infect Dis.

[35] You HL, Chang SJ, Yu HR, Li CC, Chen CH, Liao WT (2017). Simultaneous detection of respiratory syncytial virus and human metapneumovirus by one-step multiplex real-time RT-PCR in patients with respiratory symptoms. BMC Pediatr.

[36] Sugimoto S, Kawase M, Suwa R (2023). Development of a duplex real-time RT-PCR assay for the detection and identification of two subgroups of human metapneumovirus in a single tube. J Virol Methods.

[37] Feng ZS, Zhao L, Wang J (2018). A multiplex one-tube nested real time RT-PCR assay for simultaneous detection of respiratory syncytial virus, human rhinovirus and human metapneumovirus. Virol J.

[38] Xu X, Cai L, Liang S (2023). Digital microfluidics for biological analysis and applications. Lab Chip.

[39] Huang H, Huang K, Sun Y (2022). A digital microfluidic RT-qPCR platform for multiple detections of respiratory pathogens. Micromachines (Basel).

[40] Flores-Contreras EA, Carrasco-González JA, Linhares DC (2023). Emergent molecular techniques applied to the detection of porcine viruses. Vet Sci.

[41] Song Q, Zhu R, Sun Y, Zhao L, Wang F, Deng J, Qian Y (2014). Identification of human metapneumovirus genotypes A and B from clinical specimens by reverse transcription loop-mediated isothermal amplification. J Virol Methods.

[42] Wang X, Zhang Q, Zhang F (2012). Visual detection of the human metapneumovirus using reverse transcription loop-mediated isothermal amplification with hydroxynaphthol blue dye. Virol J.

[43] Wang D, Wang Y, Zhu K, Shi L, Zhang M, Yu J, Liu Y (2019). Detection of cassava component in sweet potato noodles by real-time loop-mediated isothermal amplification (Real-time LAMP) method. Molecules.

[44] Pan J, Zeng M, Zhao M, Huang L (2023). Research progress on the detection methods of porcine reproductive and respiratory syndrome virus. Front Microbiol.

[45] Li X, Zhong J, Li H (2023). Advances in the application of CRISPR-Cas technology in rapid detection of pathogen nucleic acid. Front Mol Biosci.

[46] Makarova KS, Aravind L, Wolf YI, Koonin EV (2011). Unification of Cas protein families and a simple scenario for the origin and evolution of CRISPR-Cas systems. Biol Direct.

[47] Yang R, Zhao L, Wang X, Kong W, Luan Y (2024). Recent progress in aptamer and CRISPR-Cas12a based systems for non-nucleic target detection. Crit Rev Anal Chem.

[48] Lei L, Liao F, Tan L (2022). Lamp coupled CRISPR-Cas12a module for rapid, sensitive and visual detection of porcine Circovirus 2. Animals (Basel).

[49] Zhao Z, Wang S, Dong Z, Fan Q, Lei R, Kuang R, Zhang Y (2023). One-step reverse-transcription recombinase-aided amplification CRISPR/Cas12a-based lateral flow assay for fast field screening and accurate differentiation of four major tobamoviruses infecting tomato and pepper. J Agric Food Chem.

[50] Li Y, Wang X, Xu R, Wang T, Zhang D, Qian W (2023). Establishment of RT-RPA-Cas12a assay for rapid and sensitive detection of human rhinovirus B. BMC Microbiol.

[51] Qian W, Huang J, Wang T, He X, Xu G, Li Y (2021). Visual detection of human metapneumovirus using CRISPR-Cas12a diagnostics. Virus Res.

[52] Zhao J, Ragupathy V, Liu J (2015). Nanomicroarray and multiplex next-generation sequencing for simultaneous identification and characterization of influenza viruses. Emerg Infect Dis.

[53] Gu W, Miller S, Chiu CY (2019). Clinical metagenomic next-generation sequencing for pathogen detection. Annu Rev Pathol.

[54] Tan L, Yao J, Yang Y, Luo W, Yuan X, Yang L, Wang A (2021). Current status and challenge of pseudorabies virus infection in China. Virol Sin.

[55] Tollefson SJ, Cox RG, Williams JV (2010). Studies of culture conditions and environmental stability of human metapneumovirus. Virus Res.

[56] Jeong S, Park MJ, Song W, Kim HS (2020). Advances in laboratory assays for detecting human metapneumovirus. Ann Transl Med.

[57] Keske Ş, Gümüş T, Köymen T, Sandıkçı S, Tabak L, Ergönül Ö (2019). Human metapneumovirus infection: Diagnostic impact of radiologic imaging. J Med Virol.

[58] Ma S, Zhu F, Xu Y (2023). Development of a novel multi-epitope mRNA vaccine candidate to combat HMPV virus. Hum Vaccin Immunother.

